# Fetal alcohol spectrum disorder: development of consensus referral criteria for specialist diagnostic assessment in Australia

**DOI:** 10.1186/1471-2431-14-178

**Published:** 2014-07-08

**Authors:** Rochelle E Watkins, Elizabeth J Elliott, Amanda Wilkins, Jane Latimer, Jane Halliday, James P Fitzpatrick, Raewyn C Mutch, Colleen M O’Leary, Lucinda Burns, Anne McKenzie, Heather M Jones, Janet M Payne, Heather D’Antoine, Sue Miers, Elizabeth Russell, Lorian Hayes, Maureen Carter, Carol Bower

**Affiliations:** 1 Telethon Kids Institute, The University of Western Australia, Perth, Australia; 2 Discipline of Paediatrics and Child Health, Sydney Medical School, University of Sydney, Sydney, Australia; 3 The Children’s Hospital at Westmead, Sydney, Australia; 4 The George Institute for Global Health, Sydney Medical School, University of Sydney, Sydney, Australia; 5 Department of Health Western Australia, Child and Adolescent Health Service, Perth, Australia; 6 Public Health Genetics, Genetic Disorders, Murdoch Childrens Research Institute, Melbourne, Australia; 7 Centre for Population Health Research, Curtin University, Perth, Australia; 8 National Drug and Alcohol Research Centre, University of New South Wales, Sydney, Australia; 9 Menzies School of Health Research, Charles Darwin University, Darwin, Australia; 10 National Organisation for Fetal Alcohol Spectrum Disorders, Adelaide, Australia; 11 Russell Family Fetal Alcohol Disorders Association, Cairns, Australia; 12 Centre for Chronic Disease, School of Medicine, University of Queensland, Brisbane, Australia; 13 Nindilingarri Cultural Health Services, Fitzroy Crossing, Australia

**Keywords:** Fetal alcohol spectrum disorder, Referral, Consensus

## Abstract

**Background:**

Fetal alcohol spectrum disorder (FASD) is known to be under-recognised in Australia. The use of standard methods to identify when to refer individuals who may have FASD for specialist assessment could help improve the identification of this disorder. The purpose of this study was to develop referral criteria for use in Australia.

**Method:**

An online survey about FASD screening and diagnosis in Australia, which included 23 statements describing criteria for referral for fetal alcohol syndrome (FAS) and FASD based on published recommendations for referral in North America, was sent to 139 health professionals who had expertise or involvement in FASD screening or diagnosis. Survey findings and published criteria for referral were subsequently reviewed by a panel of 14 investigators at a consensus development workshop where criteria for referral were developed.

**Results:**

Among the 139 health professionals who were sent the survey, 103 (74%) responded, and 90 (65%) responded to the statements on criteria for referral. Over 80% of respondents agreed that referral for specialist evaluation should occur when there is evidence of significant prenatal alcohol exposure, defined as 7 or more standard drinks per week and at least 3 standard drinks on any one day, and more than 70% agreed with 13 of the 16 statements that described criteria for referral other than prenatal alcohol exposure. Workshop participants recommended five independent criteria for referral: confirmed significant prenatal alcohol exposure; microcephaly and confirmed prenatal alcohol exposure; 2 or more significant central nervous system (CNS) abnormalities and confirmed prenatal alcohol exposure; 3 characteristic FAS facial anomalies; and 1 characteristic FAS facial anomaly, growth deficit and 1 or more CNS abnormalities.

**Conclusion:**

Referral criteria recommended for use in Australia are similar to those recommended in North America. There is a need to develop resources to raise awareness of these criteria among health professionals and evaluate their feasibility, acceptability and capacity to improve the identification of FASD in Australia.

## Background

There are known limitations in the recognition and diagnosis of fetal alcohol spectrum disorder (FASD) in Australia and elsewhere, with the diagnosis of FASD often delayed or missed [1]. Estimates of the prevalence of fetal alcohol syndrome (FAS) in Australia (0.6 - 0.7 per 1000 live births [2-4]), are low compared with international estimates. The prevalence of FAS in the United States is estimated to be at least 2–7 per 1000 population, and the prevalence of FASD among school children in the United States and some Western European countries is estimated to be as high as 2-5% [5]. High risk population subgroups have also been identified, including Indigenous populations and those in correctional and out of home care settings [3,6-8].

The varied nature of FASD presentations with respect to both dysmorphology and neuropsychological profile, and the lack of valid and reliable screening tests likely contribute to poor awareness of FASD as a possible differential diagnosis, uncertainty about the need for specialist assessment, infrequent referral for specialist assessment, and underidentification of this disorder. Strategies to increase recognition and diagnosis include training to improve awareness of FASD among health professionals [9,10] and establishment of dedicated clinical services for diagnosis and management [11]. However, the lack of screening tools for FASD that are specific and sensitive to prenatal alcohol exposure [12] contributes to uncertainty and inconsistency in the methods used for screening and referral [13].

Both the Canadian diagnostic guidelines for FASD [12] and the United States Centers for Disease Control and Prevention (CDC) diagnostic guidelines for FAS [14] attempt to address these uncertainties through the publication of criteria for referral. These criteria provide guidance for health professionals on when to refer individuals with specific presentations suggestive of FAS or FASD for specialist diagnostic evaluation. Criteria for referral are intended to provide clinicians with guidance relevant to routine clinical decision-making outside the formal screening context, in the absence of suitable valid and reliable formal screening tests.

There are similarities between the Canadian [12] and CDC [14] criteria for referral. Both recommend referral where there is confirmed significant prenatal alcohol exposure, which is defined in the CDC guidelines as 7 or more standard drinks per week, or 3 or more standard drinks on multiple occasions [14]. In contrast, the largest interdisciplinary diagnostic program in Washington State [15] uses any level of confirmed prenatal alcohol exposure as the main criterion for acceptance into their diagnostic assessment program. In lieu of information on confirmed prenatal alcohol exposure, the presence of a full FAS facial phenotype, which is highly specific to prenatal alcohol exposure [16-18], is also used as a criterion for referral in the CDC guidelines, Canadian guidelines and Washington State diagnostic program. The Canadian guidelines also recommend referral in the presence of probable significant prenatal alcohol exposure if at least one characteristic FAS facial anomaly and an additional diagnostic feature (growth deficit or central nervous system abnormality) are present. In contrast, the CDC guidelines include additional criteria for referral that do not require information on prenatal alcohol exposure, including the presence of a facial anomaly and growth deficit or central nervous system deficit, and parent or caregiver concern that their child might have FAS.

There is no formal guidance for Australian health professionals about when it is appropriate to refer individuals for a specialist diagnostic assessment when FASD is suspected. Surveys of paediatricians and other health professionals indicate poor awareness of these conditions and their diagnostic criteria, and a need for information and resources to improve identification [10,19]. Australian health professionals have indicated their need for standard guidelines and explicit criteria that identify how to assess individuals who may have FASD and determine when a specialist diagnostic evaluation is required [20]. This support for the development of standard criteria for referral has been highlighted in recent consensus recommendations for FASD screening and diagnosis in Australia [21].

We have been unable to locate any formal evaluation of published criteria for referral, although in the Washington State program over 90% of referred individuals receive a FASD diagnosis [15]. The lack of clear empirical evidence to inform clinical decision-making in many areas of health care has motivated researchers and policy makers to use expert judgement and consensus to develop clinical tools and practice guidelines [22-25]. Formal consensus methods enable a wide range of knowledge and experience to be considered in the criteria development process, and provide a systematic approach to evaluate and integrate multiple sources of evidence [26] and deal with uncertainty [23]. Due to the lack of formal evidence on the effectiveness of published referral criteria for FASD, we aimed to develop consensus-based referral criteria to facilitate appropriate referral practices and improved identification of FASD in Australia.

## Methods

A consensus development workshop was used to review evidence relevant to the development of locally appropriate consensus criteria for referral. Two main sources of evidence were considered: i) a published systematic review of the literature on FASD screening and diagnosis [27], updated to include literature published up to September 2010; and ii) findings from a survey of health professionals.

### Survey of health professionals

A sample of 130 Australian and 9 international health professionals was surveyed to evaluate agreement with criteria for referral that were derived from published criteria and identify perceptions relevant to the development of criteria for referral in Australia. This survey was conducted as part of a larger survey on the screening and diagnosis of FASD in Australia, and survey methods are reported in detail elsewhere [28].

### Recruitment

Health professionals with expertise or experience in FASD screening or diagnosis were invited to participate. Participants were recruited from three sources: medical practitioners who had previously reported a case of FAS to the Australian Paediatric Surveillance Unit (APSU) (n = 57) [3]; health professionals who were identified by the study investigators as having experience or expertise in FASD screening or diagnosis, including international experts in the field, based on the recommendation of consumer and community participants (n = 128); and health professionals who responded to canvassing of health professional organisations calling for individuals with relevant expertise (n = 35). Individuals passively enrolled via the APSU were advised of the study prior to survey administration, and were only removed from the panel if they declined to participate (17/57). In contrast, participants who were recruited via the steering group or professional organisations had all actively indicated their intention to participate. Consistent with these differences in recruitment, individuals who were passively recruited via the APSU were less likely to respond to the survey (67.5%) compared with participants who were recruited through professional bodies (71.0%) or the study steering group (79.4%). Of the 220 individuals invited to participate in the survey, 81 either did not respond to the email invitation or declined to participate prior to the survey administration and were excluded from the study. The survey was administered to 139 individuals who agreed to participate in the study.

### Survey development

Three published criteria for referral intended for use in the general population were identified in the systematic literature review. These included the Canadian guidelines for the diagnosis of FASD [12,29], and the CDC guidelines for the diagnosis of FAS [14], and referral criteria used by the Washington State Fetal Alcohol Syndrome Diagnostic and Prevention Network [15]. The main elements of these published criteria for referral are summarised in Table 1.

**Table 1:** Summary of published criteria for referral for individuals who may have fetal alcohol syndrome or fetal alcohol spectrum disorder by criteria content^*^

**Criterion main content**	**Chudley **[12]	**Loock **[29]	**CDC **[14]	**Astley **[15]
1. Any confirmed prenatal alcohol exposure (PAE)	-	-	-	Yes
2. Confirmed significant PAE†	Yes	Yes	Yes	-
3. 3 characteristic FAS facial anomalies^‡^	Yes	-	Yes	Yes
4. PAE (significant) and 3 characteristic FAS facial anomalies^‡^	-	Yes	-	-
5. PAE (known or probable significant) and 1 facial anomaly^‡^ and growth deficit	Yes	Yes	-	-
6. PAE (known or probable significant) and 1 facial anomaly^‡^ and CNS deficit	Yes	Yes	-	-
7. PAE (known or probable significant) and CNS deficit	-	Yes	-	-
8. 1 facial anomaly^‡^ and growth deficit and CNS deficit	-	Yes	Yes	-
9. 1 facial anomaly^‡^ and growth deficit	-	-	Yes	-
10. 1 facial anomaly^‡^ and CNS deficit	-	-	Yes	-
11. Concern by parent or caregiver that their child might have FAS	-	-	Yes	-

CDC – United States Centers for Disease Control and Prevention criteria for referral for Fetal Alcohol Syndrome.

CNS – central nervous system.

PAE – prenatal alcohol exposure.

FAS – fetal alcohol syndrome.

^†^definitions of significant or high risk exposure vary between publications: CDC guidelines define significant as exposure to 7+ standard drinks per week, or 3 or more drinks on multiple occasions, or both.

^‡^Characteristic FAS facial anomalies - smooth philtrum, thin vermillion border and small palpebral fissures.

‘-‘ not included.

^*^There is some overlap between criteria listed due to slight variations in content.

We designed 23 statements to evaluate agreement with a range of published criteria for referral (Table 2). Participants were asked to rate their agreement with each statement on a 5-point Likert scale which ranged from ‘strongly agree’ to ‘strongly disagree’. Participants were able to select ‘no comment’ if they believed that a statement was outside their area of expertise. Three open ended questions were also used to elicit opinions on criteria for referral, including the identification of alternative criteria. Pretesting was performed with 16 clinicians and researchers to test the online format and assess the clarity and face validity of the survey.

**Table 2:** Statement ratings: criteria for conducting a full diagnostic evaluation

**Statements**	**N**	**% Agree (IQD)**
***Prenatal alcohol exposure criteria: What level of alcohol exposure, at any time during pregnancy, would alone be sufficient to indicate the need for a full diagnostic evaluation for FASD:***		
Q1. Less than 7 standard drinks per week, and no more than 2 standard drinks on any one day	80	37.5 (2)
Q2. Less than 7 standard drinks per week, and between 3 and 4 standard drinks on any one day	78	61.5 (1)
Q3. 7 or more standard drinks per week, and no more than 2 standard drinks on any one day	79	59.5 (2)
Q4. 7 or more standard drinks per week, and between 3 and 4 standard drinks on any one day	81	**81.5 (1)**^1^
Q5. Binge drinking (5 or more standard drinks per occasion) less than once per week	84	**78.6 (1)**^1^
Q6. Binge drinking (5 or more standard drinks per occasion) once or twice per week	83	**84.3 (1)**^2^
Q7. No level of prenatal alcohol exposure is alone sufficient to indicate the need for a full diagnostic evaluation for FASD	72	45.8 (3)
***Other criteria: In the absence of other known causes, a full diagnostic evaluation for FASD is required when there is evidence of:***		
Q8. Concern by a parent or foster parent that their child might have a FASD	88	**88.6 (1)**^1^
Q9. All 3 of the characteristic FAS facial anomalies (smooth philtrum, thin vermillion border, and small palpebral fissures)	83	**95.2 (1)**^2^
Q10. 2 of the characteristic FAS facial anomalies	78	**76.9 (1)**^1^
Q11. The characteristic pattern of FAS facial anomalies (number unspecified)	79	**72.2 (2)**^2^
Q12. 2 of the characteristic FAS facial anomalies, **and** a growth deficit **or** any CNS abnormality (structural, neurological or functional)	80	**93.8 (1)**^2^
Q13. 2 of the characteristic FAS facial anomalies, **and** a growth deficit **and** any CNS abnormality	82	**92.7 (1)**^2^
Q14. 1 of the characteristic FAS facial anomalies, **and** a growth deficit **or** any CNS abnormality	81	67.9 (1)
Q15. 1 of the characteristic FAS facial anomalies, **and** a growth deficit **and** any CNS abnormality	81	**85.2 (1)**^1^
Q16. Known or probable prenatal alcohol exposure, **and** 1 of the characteristic FAS facial anomalies, **and** a growth deficit **or** any CNS abnormality	83	**92.8 (1)**^1^
Q17. Known or probable prenatal alcohol exposure, **and** 1 of the characteristic FAS facial anomalies, **and** a growth deficit **and** any CNS abnormality	82	**96.3 (1)**^2^
Q18. Growth deficit **and** any CNS abnormality	79	55.7 (1)
Q19. Known or probable prenatal alcohol exposure, **and** growth deficit **and** any CNS abnormality	83	**94.0 (1)**^2^
Q20. Known or probable prenatal alcohol exposure, **and** any CNS abnormality	82	**87.8 (1)**^1^
Q21. 2 or more CNS abnormalities	73	43.8 (2)
Q22. Known or probable prenatal alcohol exposure, **and** 2 or more CNS abnormalities	82	**95.1 (1)**^2^
Q23. Known or probable prenatal alcohol exposure, **and** 1 or more birth defects	81	**87.7 (1)**^1^
***Other statement about the use of the criteria:***		
Q24. A full diagnostic evaluation for FASD should occur outside standard criteria when health professionals have concerns or doubts about FASD screening results	75	**82.7 (1)**

IQD: inter-quartile deviation.

Results for statements that reached consensus agreement (≥70% agree) are presented in bold.

^1^Statement defined minimum consensus criteria for referral at the 70% level of consensus.

^2^Statement did not define minimum consensus criteria for referral at the 70% level of consensus.

A standard drink is defined as containing 10 g of alcohol [36].

### Survey administration

The survey was administered online from a secure server. An email containing a personal username and password and a link to the survey website was sent to all participants. Participants were asked to respond within 14 days, and email reminders were sent approximately 7 days and 2 days prior to the survey closure. When participant telephone numbers were available, non-responders were followed up by telephone and the survey closure date extended to improve response.

### Analysis

Descriptive statistics were generated for each statement, including response frequencies, median scores and inter-quartile deviation (IQD). Consensus agreement was defined *a priori* as 70% agreement. Qualitative data were independently coded and analysed by two investigators using qualitative inductive content analysis methods [30,31]. Data from each open ended question were reviewed alongside the quantitative data and coded inductively based on the underlying meaning of the responses. Both analysts’ coding schemes were reviewed for consistency to ensure the credibility and trustworthiness of the analysis process [30].

### Consensus development workshop

A consensus development workshop was held to develop recommendations for FASD screening, referral and diagnosis in Australia. Workshop methods and general recommendations, including criteria for diagnosis, are described in detail elsewhere [21].

### Participants

A panel of investigators with expertise in FASD was formed to conduct the study, review the findings of the systematic review and health professional survey, and develop consensus criteria for referral in Australia. Panel members included paediatricians and other health professionals, health researchers and consumer and community representatives, and three of the panel members were Indigenous. Panel members met monthly by teleconference prior to the face-to-face workshop to provide input on study design, and to review and oversee the collection and evaluation of evidence to be considered in recommendation development. Only 13 of the 17 panel members were able to attend the 2-day workshop at which consensus criteria for referral were developed; however, all panel members participated in the review of workshop outcomes and subsequent final recommendation development.

### Process

The nominal group technique [32,33] was used to structure the group process, in combination with review of evidence and open group discussion. Facilitated open group discussion enabled consensus on referral criteria to be achieved through the clarification, comparison and revision of proposed criteria for referral. Following the workshop a subgroup of the panel with specific expertise in diagnosis reviewed the workshop outcomes and confirmed the criteria to be recommended. Field notes were used to record the content of group discussions.

### Analysis

Qualitative descriptive analysis [34] of participant contributions in open discussion sessions, based on identifying and categorising the underlying meaning of participant contributions and statements [31], was used to describe the main discussion content. All participants checked the description of findings for consistency and accuracy. This research adheres to the RATS guidelines for qualitative research [35], and this study was approved by The University of Western Australia Human Research Ethics Committee and the Western Australian Aboriginal Health Information and Ethics Committee.

## Results

### Survey findings

Of the 139 individuals who were sent the survey, 103 (74%) returned a partially or fully completed survey, and 90 (65%) responded to 1 or more of the statements on referral criteria. Respondents to the statements on referral criteria were paediatricians (42%), other medical practitioners (26%) and other health professionals (32%), which included allied health professionals, midwives, nurses, health workers and health researchers. Almost three quarters of respondents (74%) were female and most (92%) were Australian. International respondents came from New Zealand (4), the United Kingdom (1) and the United States (2). Responses of the 7 international participants did not differ substantially from those of the 83 Australian participants, and due to the small number of international participants, findings are reported collectively. Over three quarters of respondents (77%) reported experience in FASD screening or diagnosis.

### Criteria for prenatal alcohol exposure

There was consensus agreement that referral for a diagnostic evaluation should occur when there is evidence of significant prenatal alcohol exposure, defined as at least 7 or more standard drinks (defined as containing 10 g of alcohol [36]) per week with 3 or 4 drinks on any one occasion, or consumption of 5 or more drinks on any one occasion (Table 2: Q4 - Q6). Approximately 60% of respondents agreed that 7 or more drinks per week, or less than 7 drinks per week, but at least 3 drinks on any one day, indicate the need for diagnostic evaluation (Table 2: Q2 and Q3).

Among the 29 survey participants who commented on the referral criteria for prenatal alcohol exposure, 9 indicated support for referral based on any level of confirmed prenatal alcohol exposure. Reasons provided included: i) a safe level of exposure has not been established, and it is difficult to define the relevant level of exposure; and ii) the use of a high level of exposure as a criterion for evaluation sends an inappropriate message to the public that lower levels of exposure are safe.

‘… that is the only way that there will be a shift in public opinion of the level of drinking that may “cause a problem”…’

‘… teratogenicity has not been defined in terms of amount of exposure or frequency of exposure, or even timing of exposure. Certainly it may be appropriate to target more at risk children if mothers have drunk greater amounts or more frequent amounts of alcohol, but this may miss a large amount of children who, whilst they may not have FAS, will fall elsewhere on the spectrum.’

Four respondents noted that detailed information on exposure is often not available, the accuracy of reports cannot be confirmed and many underreport consumption due to fear or embarrassment.

‘It would be near impossible to gauge this level of information from the clients I work with.’

‘The problem however is you will NEVER know when the exposure pattern is being accurately recalled/reported to you.’

In contrast, 7 respondents commented that evidence of heavy alcohol use or a history of alcohol-related illness or dependency should be required to indicate the need for diagnostic evaluation as well as assessment and support for the mother. An additional five respondents believed that heavy prenatal alcohol exposure is alone not sufficient to warrant diagnostic evaluation due to the limited diagnostic capacity in Australia and that referral should be based on the child’s difficulties.

‘Only higher levels of intake have consistent evidence for harm. Additional concern needed before full diagnostic work up of lesser exposures.’

‘Heavy alcohol [use] should prompt careful looking at the child, but not diagnostic evaluation.’

‘… [prenatal alcohol exposure] alone is not enough to warrant a full diagnostic evaluation. Living in an Indigenous community this criteria would indicate most of the children would need evaluations and there just aren’t the resources available for that.’

### Other criteria

Consensus agreement was reached on 13 of the 16 other criteria for referral assessed, as well as when health professionals had concerns or doubts about screening results (Table 2). Among the 14 participants who commented on the other criteria for referral assessed, general comments about the criteria for referral indicated: support for sensitive criteria for referral to reduce the risk of missing cases; that diagnostic evaluations for FASD should not stand alone and are part of an integrated assessment process for neurological or developmental concerns; that the criteria for parental concern is too non-specific; that the distinction between screening and diagnosis is unclear; and that assessment is required to rule out other known causes of the criteria for referral, with FASD possibly a differential diagnosis.

‘… FASD can present in any of these combinations because of its spectrum nature. All combinations might therefore be of relevance, along with other clinical context information needed to weigh them in any particular case.’

‘… concerns by a carer are not sufficient on their own to warrant a full diagnostic assessment. However, the child should undergo screening.’

### Workshop recommendations

Workshop participants aimed to identify a small number of referral criteria that: can be applied in the case of both known and unknown prenatal alcohol exposure; have a specificity comparable with previously published criteria; and exclude criteria which are not consistent with the diagnostic categories recommended for use in Australia (FAS, partial FAS (PFAS) and neurodevelopmental disorder-alcohol exposed (ND-AE) [21]).

Referral criteria endorsed by survey respondents at the 70% consensus level were less specific in some aspects than criteria included in published guidelines, including agreement with referral based on a ‘characteristic pattern of FAS facial anomalies (number unspecified)’. Workshop participants recommended the use of more specific criteria for referral based on facial anomalies, and that parental or caregiver concern should prompt assessment against the criteria for referral and monitoring of growth and development if referral is not indicated at that time.

Five independent criteria for referral were recommended (Table 3), with referral considered appropriate if individuals satisfied any one of the five criteria. The inclusion of a criterion for referral based on confirmed prenatal alcohol exposure alone was most contentious, with strong debate among panel members about the ability to identify and assess an appropriate level of prenatal alcohol exposure sufficient to warrant referral. The absence of evidence for a minimum safe level of exposure to alcohol during pregnancy [37,38] was contrasted with strong evidence linking adverse outcomes with moderate or high-level exposure [39-42]. It was noted that the factors that mediate prenatal alcohol-related harm are not fully understood, and factors that influence the ability to report relevant exposure and the existing capacity for service delivery must be considered in the development of criteria for referral. Panel members agreed that referral based on prenatal alcohol exposure alone should be limited to confirmed moderate or high level exposure, similar to criteria in the Canadian and CDC guidelines and consistent with the survey findings.

**Table 3:** Recommended Australian criteria for referral and their correspondence with published criteria

**Recommended Australian criteria for referral**		**Equivalent Criteria**		
	**Chudley **[12]	**Loock **[29]	**CDC **[14]	**Astley **[15]
1. Confirmed significant^†^ prenatal alcohol exposure	Yes	Yes	Yes	-
2. 3 Characteristic FAS facial anomalies^‡^	Yes	-^#^	Yes	Yes
3. 1 Facial anomaly^‡^ and growth deficit and 1 or more CNS abnormalities	-	Yes	Yes	-
4. Microcephaly and any confirmed prenatal alcohol exposure	-	Yes^*^	-	-
5. 2 or more CNS abnormalities and any confirmed prenatal alcohol exposure	-	Yes^*^	-	-

CDC – United States Centers for Disease Control and Prevention criteria for referral for Fetal Alcohol Syndrome.

CNS – central nervous system.

^†^significant exposure was defined as exposure to: 7+ standard drinks per week, or 5+ standard drinks on any one occasion.

^‡^Characteristic FAS facial anomalies - smooth philtrum, thin vermillion border and small palpebral fissures.

^#^guideline also requires evidence of significant prenatal exposure to alcohol.

^*^equivalent original criteria includes CNS deficits and known or probable significant PAE [29].

Two referral criteria were developed which do not require the presence of confirmed prenatal alcohol exposure (Table 3, criteria 2 and 3), and which were intended to facilitate the referral of individuals who may have FAS. Despite initial support for referral based on the presence of two characteristic FAS facial anomalies alone, which diverges from criteria included in all other guidelines, participants ultimately agreed to recommend referral based on the presence of all three characteristic FAS facial anomalies. The simultaneous presence of all three facial anomalies is specific to prenatal alcohol exposure [16-18], and provides a mechanism for referral in the absence of information on prenatal alcohol exposure. Panel members noted that relaxation of the criteria for facial anomalies has been associated with lack of specificity for prenatal alcohol exposure and CNS dysfunction [43].

The potential unreliability of palpebral fissure length measurement by inexperienced assessors was a consideration in criteria development. However, given the inclusion of alternative criteria for referral for individuals with unknown prenatal alcohol exposure, the need to assess all three facial anomalies was anticipated to be infrequent. These alternative criteria, which were endorsed by survey respondents (Table 2 Q14 and Q18) and workshop participants, require the presence of a single facial anomaly, growth deficit at any time, and CNS abnormality. This need for evidence of abnormality in three core areas in the absence of information on prenatal alcohol exposure is present in both the CDC guidelines for FAS [14] and the Canadian guidelines for FASD [29] (Table 3).

The two final recommended criteria for referral provide a mechanism for the referral of individuals with possible ND-AE based on evidence of any confirmed prenatal alcohol exposure in conjunction with significant CNS abnormalities. Panel members noted the significance of microcephaly among individuals with FASD [44], with a prevalence of 45% among individuals with diagnosed FAS and PFAS, and 25% among individuals diagnosed with static encephalopathy-alcohol exposed using University of Washington criteria [15].

Evaluation of the consensus referral criteria was recommended to ensure that the criteria can effectively identify individuals at high risk of FASD. Guidelines and training resources for health professionals to support the implementation of these criteria, and resources for individuals referred for diagnosis, were also recommended.

### Assessment methods

Panel members recommended assessment in the following four main areas.

### Prenatal alcohol exposure

A detailed history of alcohol consumption during pregnancy is not always available. Information from medical or other official records, or from a reliable witness, may be required to establish direct evidence of confirmed prenatal alcohol exposure or alcohol dependency during the index pregnancy. Where an exposure history can be obtained, assessment of the level of known prenatal alcohol exposure should include the Alcohol Use Disorders Identification Test-Consumption (AUDIT-C) questions [45] consistent with nationally recommended assessment methods [46]. The context and timing of exposure to alcohol and other potentially teratogenic substances during pregnancy should also be assessed, including exposure prior to confirmation of pregnancy. Assessment at birth should include signs of alcohol withdrawal in the mother and neonate.

### Characteristic FAS facial anomalies

**Figure 1:**
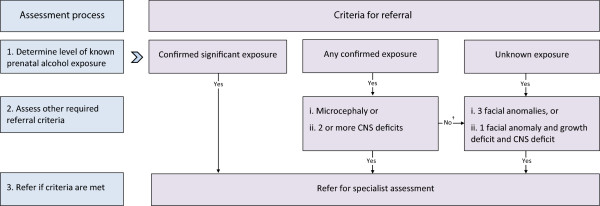
**Summary of recommended 3-step referral assessment process and criteria for individuals who may have fetal alcohol spectrum disorder.** †If other required criteria for any confirmed exposure are negative, assess criteria for unknown exposure CNS – central nervous system.

Assessment of facial anomalies is not required in all referral scenarios; however, it has particular significance where prenatal alcohol exposure is unknown (Figure 1). Assessment for the thin vermillion border and smooth philtrum characteristics of FAS should be conducted using the appropriate University of Washington Lip-Philtrum Guide [47]. Ranks 4 and 5 are considered consistent with FAS. Ideally, measurement of palpebral fissure length should be performed by trained and experienced assessors as described in the University of Washington 4-Digit Diagnostic Code [47], because physical measurements of palpebral fissure length by inexperienced assessors may be unreliable. Where clinicians experienced in this assessment are unavailable, accurate assessment of all characteristic FAS facial anomalies may be performed using facial photographic screening [17], which could be reviewed by experts locally or remotely by an external service.

### Growth

Assessment for growth deficit at any time that is unexplained by other causes should be performed using standard objective anthropometric methods and compared with locally appropriate population references for age and gender, such as the CDC growth charts [48]. Standard protocols should be used to correct for gestational age if required and parental heights if available.

### Central nervous system (CNS) abnormalities

Non-diagnostic indicators of CNS abnormality may be identified during the physical examination; through objective assessments of CNS structure or function, including the results of investigations or scans; from the results of valid and reliable behavioural and developmental screening tools, such as the Strengths and Difficulties Questionnaire [49] or from reports by parents, carers or other credible sources. A range of valid and reliable developmental and behavioural screening tools routinely used by health professionals in Australia could be used to indicate the need for referral. Different screening tests may be required for different age groups and settings. Assessment of CNS abnormalities should include measurement of head circumference and comparison with appropriate population references.

## Discussion

There is currently no international consensus on referral criteria for individuals with specific presentations suggestive of FASD for specialist diagnostic evaluation, and there is little published empirical evidence to guide recommendation development or the design of other strategies to improve recognition of these disorders. Consultation with health professionals and a consensus development workshop were used to review published criteria for referral and adapt these for the Australian context. Consistent with existing criteria for referral, we found that survey and workshop participants supported the use of indicators linked to both aetiological and diagnostic factors. Four of the five consensus criteria are similar to published Canadian criteria [29], and three include elements represented in the CDC guidelines for the diagnosis of FAS [14].

There is a recognised need for distinction between screening and diagnostic assessments for FASD. However, there is little specific information available about how recommended criteria for referral should be implemented. The proposed consensus referral criteria provide a well-defined conceptual framework for assessment which is distinct from that used to guide diagnostic assessment, despite the use of diagnostic assessment tools for growth, facial anomalies and CNS abnormalities where required. Specification of the conceptual basis for application of each referral criterion (Figure 1) enables health professionals to identify the minimum extent of assessment required to fulfil the recommended referral criteria. Depending on the degree of information available on prenatal alcohol exposure, completion of assessments for all four domains may not be required. This may be of particular value for practitioners who lack experience in the more specialised aspect of facial anomaly assessment. Individuals meeting the criteria for referral should undergo comprehensive assessment by a relevant specialist medical practitioner using a multidisciplinary assessment approach [21].

The five consensus criteria for referral are based on a 2-stage assessment process which involves assessment of prenatal alcohol exposure and identification of additional indicators when prenatal alcohol exposure in the high risk range is not confirmed. This approach in part allows for the ability of parents or caregivers to report exposures. With the intent of maintaining specificity, evidence of other abnormalities associated with prenatal alcohol exposure is required for referral where there is no evidence of prenatal exposure to alcohol in the high risk range. The presence of all three facial anomalies has particular significance as a sensitive and specific indicator of prenatal alcohol exposure [16-18], and assessment is only required in the case of unknown prenatal alcohol exposure. A range of evidence may be used in the referral context to establish the presence of CNS abnormality. A comprehensive assessment of CNS function may be utilised if available, but is not required prior to the diagnostic assessment.

Evidence of prenatal alcohol exposure is the most consistently used indicator of the need for referral for specialist assessment among published criteria, although there is some variation in the definition of this criterion. The agreed minimum level of exposure that alone indicates the need for specialist assessment correlates well with the CDC criteria for referral [14], ‘significant exposure at a level associated with physical or developmental effects’ described in the Canadian guidelines [12], and evidence on the potential importance of moderate levels of prenatal alcohol exposure to fetal behavioural outcomes [50]. Less than 40% of survey participants agreed that individuals who were exposed to less than 7 standard drinks per week and no more than 2 drinks on any one occasion should undergo specialist assessment in the absence of other indicators.

The consensus criterion for high risk exposure is more conservative than the criterion of any confirmed prenatal alcohol exposure used in the Washington State program [15]. Although the decision was not unanimous, panel members reached consensus that any confirmed prenatal alcohol exposure alone as an indicator for referral was too nonspecific, and that any prenatal exposure should only indicate the need for referral in combination with evidence of CNS abnormalities. Evaluation of referrals for the Washington State diagnostic clinics supports the validity of the use of any prenatal alcohol exposure as an indicator of FASD risk [15]. However, research indicates that most individuals assessed were exposed to high levels of prenatal alcohol exposure or had an alcohol use disorder [15], and factors that influence the implementation of this criterion, including whether the presence of other features influence the decision to refer, have not been described. The levels of exposure considered to be associated with a high risk of FASD will require review as further evidence on the effects of lower level exposures becomes available, as noted in other guidelines [12,14,47].

We found agreement that the presence of all three characteristic FAS facial anomalies is an important indicator of the need for a referral in the absence of confirmed prenatal alcohol exposure, consistent with existing recommendations [12,14,15]. Referral based on the presence of all three facial anomalies primarily targets individuals with FAS and who have unknown prenatal alcohol exposure. These presentations are likely to be only a small proportion of eligible cases [5], and FAS is the only disorder that can be diagnosed in the absence of information on prenatal alcohol exposure due to the specificity of the three characteristic FAS facial anomalies. Although there is evidence of a correlation between the presence of characteristic FAS facial anomalies, prenatal alcohol exposure and brain dysfunction [18] which suggests that partial expressions of the FAS facial phenotype may be important risk factors for brain damage associated with prenatal alcohol exposure [51,52], there is insufficient evidence to justify relaxation of this referral criterion at this time.

Further research is required to optimise these consensus referral criteria and recommended assessment processes based on the evaluation of their feasibility, acceptability and effectiveness. The implementation of referral criteria must recognise the challenges and limitations for clinicians who have little expertise in the assessment of facial anomalies and those who work in regional and remote locations. Concerns about the use of facial dysmorphology assessment outside the diagnostic context, the potential unreliability of formal assessment of palpebral fissure length among inexperienced assessors, and the suitability of existing norms for the Australian population require investigation. The establishment of a centralised facial photographic analysis service to enable specialist assessment of facial anomalies using locally captured digital images may provide a feasible and accurate method to support health professionals evaluate the need for referral. Ultimately our recommended referral criteria aim to inform the health professionals’ clinical decision making, and uncertainty about assessment findings can also provide the basis for specialist referral based on health professional concern.

Although only 77% of respondents reported experience in FASD screening or diagnosis, expertise relevant to the development of criteria for referral is not confined to individuals with practical experience in screening or diagnosis, and includes individuals who have non-clinical roles. The established under-recognition of FASD in Australia [3] is also consistent with Australian health professionals’ limited experience in screening and diagnosis. Approximately 70% or more of survey respondents completed the questions on referral criteria, suggesting that most believed they had expertise relevant to the development of criteria for referral. Overall response to the survey was lowest among participants who were passively recruited through the APSU. Although the survey response exceeded the 70% recommended level [53] and is comparable to that reported among similar Delphi studies of health professionals [54,55], non-response may have influenced the survey findings.

The lack of empirical evidence relevant to the development of recommended Australian criteria for referral was also a limitation of this study. Recommended criteria were developed by adapting criteria included in published guidelines based on input from health professionals and review within a formal consensus development framework. Although not all panel members were able to attend the 2-day face-to-face consensus development workshop, all panel members were engaged in reviewing evidence and outputs prior to and following the workshop and in formulating the final recommendations. An understanding of the appropriateness, feasibility and performance of these criteria in the Australian context, including evaluation of their predictive value and cost-effectiveness is now required. Examination of the appropriateness of existing population reference data in culturally diverse populations in Australia is also needed. The successful implementation of referral criteria will also depend upon the ability of services providers who do not have specific expertise in FASD to recognise the issue, be aware of the specialist services available for diagnosis and management, and of the potential benefits of referral.

## Conclusion

We have established the basis for nationally applicable criteria for referral in Australia. Further work is required to evaluate the appropriateness and effectiveness of the criteria for referral, and develop resources to facilitate implementation of standard referral practices, including training and support for health professionals and information for individuals who are undergoing diagnosis and their parents or carers. These processes will support the incorporation of standard criteria for referral into cost effective strategies to improve the ability of health professionals to identify and prevent FASD. Establishing effective mechanisms for referral is critical to improving the capacity for, and access to, FASD diagnosis in Australia.

## Abbreviations

APSU: Australian paediatric surveillance unit; AUDIT-C: Alcohol use disorders identification test-consumption; CDC: United states centers for disease control and prevention; CNS: Central nervous system; FAS: Fetal alcohol syndrome; FASD: Fetal alcohol spectrum disorder; PFAS: Partial fetal alcohol syndrome; IQD: Inter-quartile deviation; PAE: Prenatal alcohol exposure.

## Competing interests

The authors declare that they have no competing interests.

## Authors’ contributions

CB, EJE and JMP designed the study and CB and EJE supervised the study. CB, EJE, REW, JL and HJ designed the study survey; CB, REW, AM and HJ designed the workshop program; and all authors were members of a project steering group that reviewed the study methods and procedures. AM and REW facilitated the workshop. REW analysed the data. REW drafted the manuscript and all authors reviewed the manuscript. All authors read and approved the final version of the manuscript.

## Pre-publication history

The pre-publication history for this paper can be accessed here:

http://www.biomedcentral.com/1471-2431/14/178/prepub
